# *Lyssavirus* [lis′ə-vi′′rəs]

**DOI:** 10.3201/eid1508.999999

**Published:** 2009-08

From the Greek *lyssa* (frenzy or madness) and Latin *virus* (poison). In Greek mythology, Lyssa was the goddess of rage, fury, and rabies, known for driving mad the dogs of the hunter Acteon and causing them to kill their master. Aristotle (4th century BCE) said, “Dogs suffer from the madness. This causes them to become irritable and all animals they bite to become diseased.” The disease in humans was characterized by hydrophobia, in which the sick person was simultaneously tormented with thirst and with fear of water. Hippocrates is believed to refer to rabies when he said that persons in a frenzy drink very little, are disturbed and frightened, tremble at the least noise, or are seized with convulsions.

Lyssavirus is a genus of the family Rhabdoviridae, which includes rabies virus and other related viruses that infect mammals and arthropods (e.g., Australian bat lyssavirus, Duvenhage virus, European bat lyssaviruses 1 and 2, Lagos bat virus).

